# Intensity modulated radiotherapy for high risk prostate cancer based on sentinel node SPECT imaging for target volume definition

**DOI:** 10.1186/1471-2407-5-91

**Published:** 2005-07-28

**Authors:** Ute Ganswindt, Frank Paulsen, Stefan Corvin, Kai Eichhorn, Stefan Glocker, Ilse Hundt, Mattias Birkner, Markus Alber, Aristotelis Anastasiadis, Arnulf Stenzl, Roland Bares, Wilfried Budach, Michael Bamberg, Claus Belka

**Affiliations:** 1 Department of Radiation Oncology, University of Tübingen, Tübingen, Germany; 2 Department of Radiation Oncology, Biomedical Physics, University of Tübingen, Tübingen, Germany; 3 Department of Urology, University of Tübingen, Tübingen, Germany; 4 Department of Nuclear Medicine, University of Tübingen, Tübingen, Germany; 5 Department of Radiation Oncology, University of Düsseldorf, Düsseldorf, Germany

## Abstract

**Background:**

The RTOG 94-13 trial has provided evidence that patients with high risk prostate cancer benefit from an additional radiotherapy to the pelvic nodes combined with concomitant hormonal ablation. Since lymphatic drainage of the prostate is highly variable, the optimal target volume definition for the pelvic lymph nodes is problematic. To overcome this limitation, we tested the feasibility of an intensity modulated radiation therapy (IMRT) protocol, taking under consideration the individual pelvic sentinel node drainage pattern by SPECT functional imaging.

**Methods:**

Patients with high risk prostate cancer were included. Sentinel nodes (SN) were localised 1.5–3 hours after injection of 250 MBq ^99m^Tc-Nanocoll using a double-headed gamma camera with an integrated X-Ray device. All sentinel node localisations were included into the pelvic clinical target volume (CTV). Dose prescriptions were 50.4 Gy (5 × 1.8 Gy / week) to the pelvis and 70.0 Gy (5 × 2.0 Gy / week) to the prostate including the base of seminal vesicles or whole seminal vesicles. Patients were treated with IMRT. Furthermore a theoretical comparison between IMRT and a three-dimensional conformal technique was performed.

**Results:**

Since 08/2003 6 patients were treated with this protocol. All patients had detectable sentinel lymph nodes (total 29). 4 of 6 patients showed sentinel node localisations (total 10), that would not have been treated adequately with CT-based planning ('geographical miss') only. The most common localisation for a probable geographical miss was the perirectal area. The comparison between dose-volume-histograms of IMRT- and conventional CT-planning demonstrated clear superiority of IMRT when all sentinel lymph nodes were included. IMRT allowed a significantly better sparing of normal tissue and reduced volumes of small bowel, large bowel and rectum irradiated with critical doses. No gastrointestinal or genitourinary acute toxicity Grade 3 or 4 (RTOG) occurred.

**Conclusion:**

IMRT based on sentinel lymph node identification is feasible and reduces the probability of a geographical miss. Furthermore, IMRT allows a pronounced sparing of normal tissue irradiation. Thus, the chosen approach will help to increase the curative potential of radiotherapy in high risk prostate cancer patients.

## Background

Treatment strategies for localised prostate cancer have to address issues of local control, prevention of distant spread and treatment of microscopic locoregional spread. It has been shown that local control is critically interrelated with the prevention of distant spread, since late metastases occur in patients with local failure [1]. In order to increase the efficacy of radiotherapy, many recent trials suggest the strategy of dose escalation [2-19].

Particularly the availability of three-dimensional conformal radiation treatment (3D-CRT) and further development of intensity modulated radiation treatment (IMRT) allow the application of higher radiation doses to the prostate without increased toxicities to normal tissues. The available data suggest that prostate cancer especially in patients with intermediate risk profile benefit from dose escalation (reviewed in [20]). On the other hand, patients with a high risk profile do not show such a steep dose response relationship because of the risks of loco- regional and ultimately systemic spread.

A further approach to increase local control rates and the rates of biochemical disease control is the combination of radiation with anti-hormonal treatment. Several recently published results of larger trials [21-23] could demonstrate a clear benefit from a combined treatment in patients with locally advanced prostate cancer. Most of these trials have employed radiation portals covering the locoregional lymphatic drainage in addition to the prostate using a standardised anatomical definition.

Of special importance in this regard is the four-armed RTOG 94-13 trial comparing neoadjuvant vs. adjuvant anti-hormonal treatment and radiotherapy of the whole pelvis vs. the prostate only. Patients with an estimated risk of pelvic lymph node involvement > 15% had a significant benefit when treated with neoadjuvant and concurrent hormonal ablation in combination with radiotherapy to the prostate (70.2 Gy) and the pelvic lymph nodes (50.4 Gy). The most significant difference in 4-year progression free survival rates was observed between the groups of whole pelvis (61%) and prostate only (45%) irradiated patients when treated with neoadjuvant and concurrent hormonal ablation [23].

Thus, besides dose escalation strategies or combination with anti-hormonal treatments, adjuvant coverage of the pelvic lymph nodes may be of importance for an optimised tumour control.

Currently, the definition of an adjuvant pelvic target volume is derived from non-individualised anatomical lymphatic drainage patterns. Radiation planning and therapy is mostly performed as 3D-CT based treatment along the ICRU 50 guidelines.

However, the lymphatic drainage from the prostate is highly variable. In this regard, data on sentinel lymph nodes analysis in prostate cancer are of key importance. To optimise lymphadenectomy techniques, Wawroschek et al. [24,25] and Weckermann et al. [26] tested the sentinel lymph node (SN) identification with prostate lymphoscintigraphy and radio-guided surgery in large cohort. The results demonstrated that sentinel node identification in prostate cancer is feasible with a sensitivity between 93 and 96% and enables higher detection rates of micrometastases. However, the authors noted a strong individual variability of the lymphatic drainage in their patient cohort. Interestingly, only 44.2% of detectable cases with positive nodes were found in the obturator fossa, the most common area for a limited lymphadenectomy. These findings correspond to surgical data of 103 patients after extended lymphadenectomy [27]. In the latter, the most common areas of lymph node metastases were the external and internal iliac, followed by obturator, common iliac and presacral regions.

Considering these results, the definition of standardised target volumes covering the pelvic lymph nodes is associated with several problems. Trying to cover all possible areas of lymphatic drainage increases the probability of toxicities. In contrast, by limiting the target volume to the most commonly involved lymph node areas increases the risk of incomplete target volume coverage.

To overcome the limitations described above, we started an IMRT trial for high risk prostate cancer with a target volume definition based on the average distribution of positive lymph nodes in prostate cancer patients complemented by sentinel node functional imaging with SPECT.

## Methods

### Patients

Patients with histologically proven high risk prostate cancer, but cN0 cM0 stage, were included after providing informed consent. High risk profile was defined as T3 or T4 stage (all) or PSA > 20 ng/mL (all) or PSA 10 – 20 ng/mL with Gleason Scores = 7. Besides PSA level, rectal-digital examination and biopsy, the pre-therapeutic staging included a computed tomographic and/or magnetic resonance imaging of the abdomen and pelvis, an ultrasound of the prostate, a total body bone scan and X-ray of the chest. The clinical TNM stage was determined based on these staging results.

### Localisation of sentinel nodes

**Figure 1:**
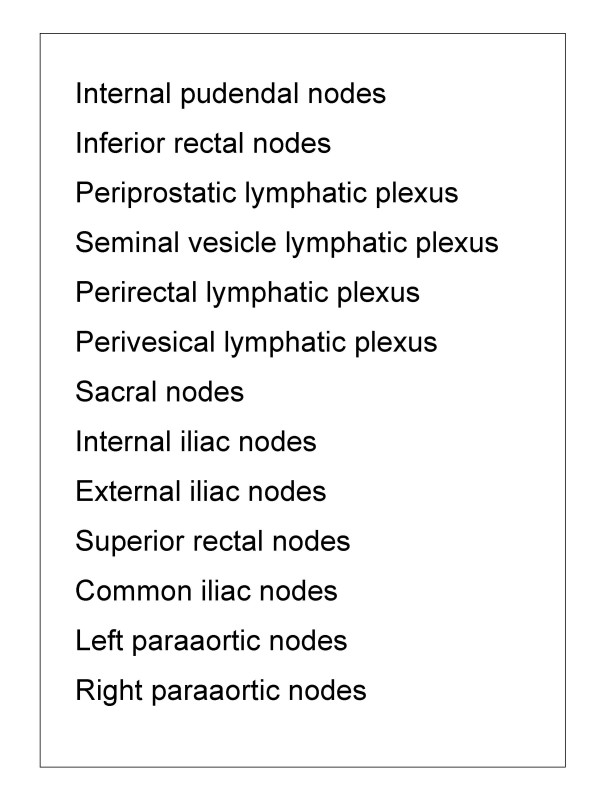
Terms and definitions of nodal groups according to Martinez-Monge et al. (1999). The obturator and hypogastric lymph nodes include periprostatic and seminal vesicle lymphatic plexus, parts of perivesical and the perirectal lymphatic plexus nearby the prostate, the ventrocranial parts of the internal pudendal and inferior rectal nodes

To permit a 3D-localisation of the sentinel nodes, transmission and emission scans were acquired using a double-headed gamma camera with an integrated X-Ray device (Millennium VG & Hawkeye^®^, GE Medical Systems Europe, Buc Cedex, France) 1.5–3 hours after transrectal intraprostatic injection of 250 MBq ^99m^Tc-Nanocoll. A single ultrasound-guided central application was performed per prostate lobe. After the anatomical-functional image fusion, the scans were analysed with respect to 3D-localisation and number of sentinel nodes. The sentinel nodes were three-dimensionally located by an experienced member of the nuclear medicine department (K.E., R.B.). For a systematic topographic evaluation of lymph node localisation, we used the Cross-sectional Nodal Atlas published by Martinez-Monge 1999 [28](figure 1).

### Treatment planning

Intensity modulated radiotherapy planning was based on three CT scans of the pelvis in supine (2 patients) and prone (4 patients, using a belly board) position at 3 mm slice spacing from two cm below the ischeal tuberosities to the L 4/5 interspace. For a comfortable bladder filling, patients were instructed to avoid urination during the last 45 minutes before the procedure. The CT datasets were transferred to the Pinnacle^® ^treatment planning system (Philips Medical Systems, DA Best, Netherlands) for segmentation. Clinical target volume (CTV) and organs at risk (OAR) were outlined in each CT image. OAR included rectum, colon/sigmoid, small bowel, bladder and hips. Image fusion was done by adjusting the bone structures of the pelvis. Finally, a single enclosing contour was derived from the three outlined CTV/OAR. For the resulting planning target volume (PTV) the enclosing CTV contour was expanded with a 3D safety margin of 7 mm.

Since two different risk areas are treated simultaneously, the term first- and second-order CTV will be used in the following. The first-order CTV included the prostate and the base of seminal vesicles. In cases of T stage ≥ 3 or an estimated risk for an involvement of the seminal vesicles > 15% (Partin nomograms) [29], seminal vesicles were included into the boost volume.

The second-order CTV included the first order CTV and routinely the obturator and hypogastric lymph nodes (following the nomenclature introduced by Martinez-Monge: Periprostatic and seminal vesicle lymphatic plexus, the parts of the perivesical and the perirectal lymphatic plexus nearby the prostate, the ventrocranial parts of the internal pudendal and inferior rectal nodes), the internal and external iliac lymph nodes (from the bifurcation of the common iliac lymph artery at the level of the upper sacroiliac joints, to the crossing point of the external iliac artery and the inguinal ligament) and the sacral nodes anterior the first and second sacral segment. In addition to this regular second-order CTV all detected sentinel node localisations were outlined separately and involved into the IMRT target volume definition.

Dose prescriptions were 50.4 Gy (5 × 1.8 Gy / week) to the pelvic nodes and 70.0 Gy (5 × 2.0 Gy / week) to the prostate and the base of seminal vesicles or whole seminal vesicles by an integrated boost planning. After forward decision regarding gantry angles, an inverse treatment planning was carried out by the Hyperion^® ^software package (University of Tübingen, Germany) [30]. Based on a class solution, six IMRT fields were used for the initial 28 fractions and 5 fields for the ensuing 7 fractions. The following normal tissue constraints were chosen: Rectum, 58–65 Gy serial dose constraint; Bladder, 50–56 Gy serial dose constraint; Small Bowel, 20–22 Gy mean dose constraint and 50–54 Gy maximum dose constraint. The dosimetrics were calculated by Monte Carlo dose calculation.

Plans were produced for a 15 MV linear accelerator (Elekta Oncology Systems^®^, Crawley, UK) for delivery with a multileaf collimator system using a step and shoot technique. The IMRT plans were compared with 3D-CRT plans (four-field-technique, gantry angles 0°, 90°, 180°, 270°) using the Pinnacle^® ^treatment planning system based on the identical CTV. Dose volume histograms (DVH) were calculated for each plan. The PTV dose ranges were calculated with the minimum PTV dose defined as dose received by ≥ 99% of the PTV and the maximum dose defined as the dose received by = 1% of the PTV. The volume percentages of small bowel, large bowel (sigmoid/colon), rectum and bladder irradiated with 63 Gy, 56 Gy, 35 Gy, 14 Gy were recorded for each technique. The DVH comparisons were done for each patient using the summarised plan.

### Verification

Prior to each radiation fraction, the isocenter was verified by portal imaging from 0° and 90° gantry angle. An error of more than 2 mm was corrected. The administered monitor units needed for one daily verification were included in the initial dose calculation.

### Analysis of toxicity

Acute gastrointestinal, genitourinary and skin toxicities (RTOG criteria) were documented by standard forms weekly during RT and at least after three months.

## Results

### Patients

**Figure 2:**
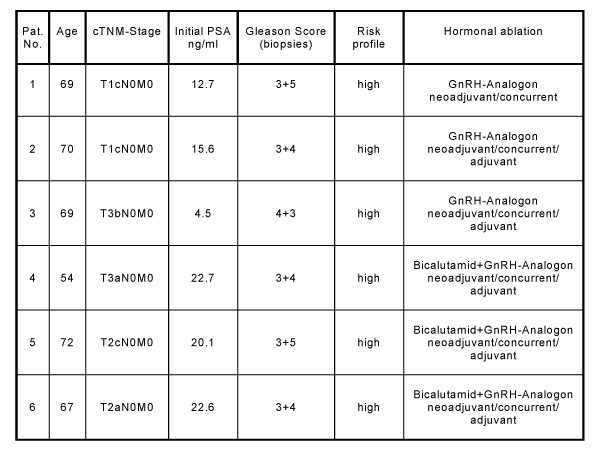
Patients characteristics.

Since August 2003, six patients with T1c-3b high risk prostate cancers (figure 2) were evaluated. No patient had undergone a staging lymphadenectomy of the pelvic lymph nodes before. All patients had normal pre-therapeutic diagnostic findings with a clinical N0 M0 stage. All patients received neoadjuvant and concurrent hormonal ablation, which was recommended to continue adjuvantly for three years. In all cases neoadjuvant treatment had been started before the first visit in hospital.

### Localisation of sentinel nodes

**Figure 3:**
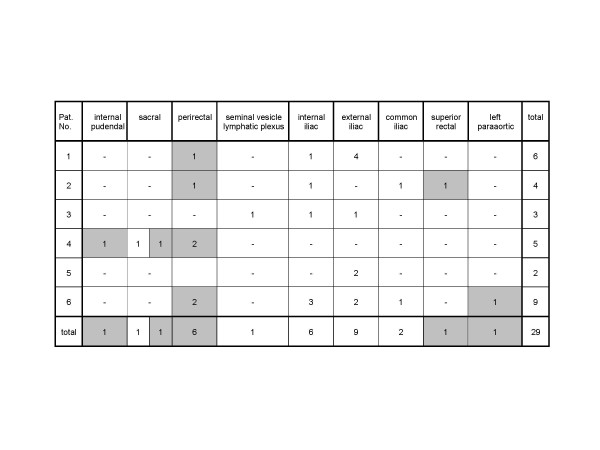
Number and localisation of sentinel nodes. Localisation of lymph node areas not covered by a standard target volume definition plan are shaded grey (geographical misses).

The transrectal intraprostatic injection of ^99m^Tc-Nanocoll was performed in all patients without any complications. After radioisotope injection and image fusion, each patient had detectable sentinel lymph nodes. The number per patient ranged from two to nine detected lymph nodes. Altogether, a total of twenty-nine lymph nodes could be identified for all six patients (figure 3). The most common localisations of identified lymph nodes were external iliac (9), followed by internal iliac (6) and perirectal lymphatic plexus (6). Further localisations were common iliac (2), sacral (2), internal pudendal (1), seminal vesicle lymphatic plexus (1), superior rectal (1) and left paraaortic (1).

### IMRT treatment planning

**Figure 4:**
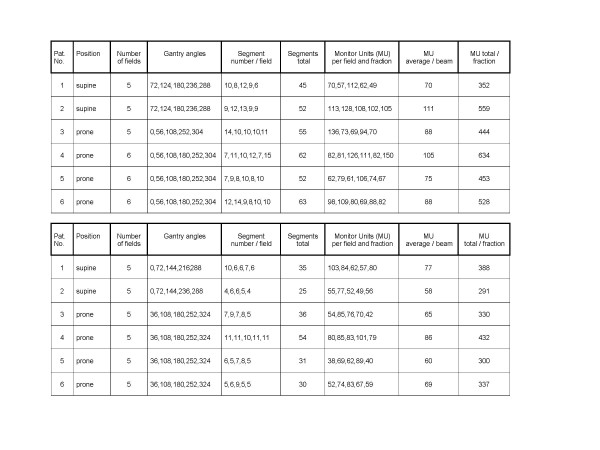
IMRT planning results: 28 fractions including pelvic nodes with integrated boost (upper table) and 7 fractions without pelvic nodes (lower table). MU total / fraction adds up MU's per field and fraction (rounded), including additionally 3 MU's necessary for daily verification (not included in 'MU's per field and fraction)

The first two patients were planned and treated in supine position. Since prone position in combination with a belly board allowed easier sparing of small bowel [31], we changed to the prone position for the following four patients. All detected sentinel lymph nodes could be included into the second-order CTV. The IMRT plans carried out by Hyperion^® ^produced an average of 55 segments (total/fraction) and 495 Monitor Units (total/fraction) for the first 28 fractions and an average of 35 segments (total/fraction) and 346 Monitor Units (total/fraction) for the ensuing 7 fractions. Thus, fast delivery and practical treatment times were possible. A class solution delivered stable and comparable results for all patients. Detailed characteristics of IMRT technique are shown in figure 4.

**Figure 5:**
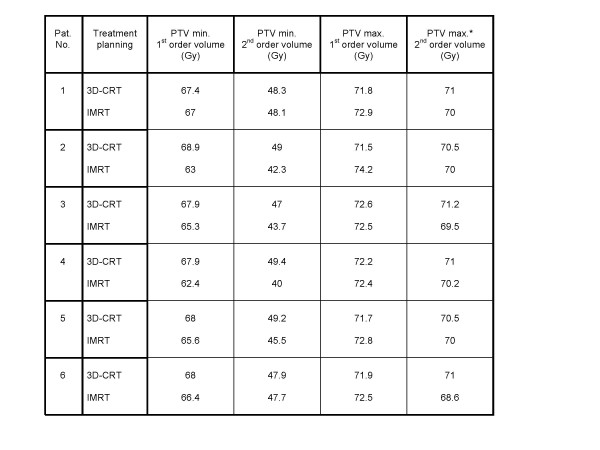
PTV dose ranges 3D-CRT vs. IMRT. Minimum PTV dose defined as the dose received by = 99% of the PTV, maximum PTV dose defined as the dose received by = 1% of the PTV. * Dose contribution by the boost RT.

The minimum PTV dose ranged from 40 to 48.1 Gy for the second-order PTV, including the pelvic nodes. The maximum PTV dose ranged from 72.4 to 74.2 Gy for the prostate gland (figure 5). The critical dose of 60 Gy to the whole bladder volume was not reached in any patient.

A critical dose to the small bowel of 50 Gy was reached in two patients in very small volumes (0.5% and 1%, respectively). Large bowel doses ≥ 50 Gy were seen in very limited volumes (0%, 2.7%, 2.7%, 4.1%, 6.6% and 13.6%). The single event of a higher dose volume of 13.6% was seen in one patient (No. 5) with an adherent sigmoid loop in close vicinity to the prostate. Rectum volumes irradiated with more than 56 Gy ranged from 16.3% to 52.1%, with more than 63 Gy from 9.5% to 36.6%. The detailed analysis of irradiated organs at risk volume percentages is given in table 6.

### Influence of sentinel node localisation on target volume definition

4 of 6 patients had sentinel node localisations (total 10 of 29 sentinel lymph nodes) that would not have been treated adequately with conventional target volume definition ('geographical miss'). Thus, the target volume definition was modified by sentinel lymph node information in 4 of 6 patients. The results of numbers and localisations of sentinel lymph nodes are shown in table 3, grey shaded fields show the number and localisation of geographical misses.

The lymph node area associated with the highest probability of a 'geographical miss' was the perirectal lymph node group with 6 identified sentinel nodes partly localised nearby the dorsal circumference of the rectal wall. Single sentinel nodes were found in the internal pudendal, sacral, superior rectal and left paraaortic region. None of those was covered by standardised planning target volumes. The identified sentinel lymph node in the left paraaortic area was localised at the L4/5 interspace. Because the CT scans showed a lymph node of 11 mm diameter at this localisation, we decided to include it.

### DVH comparison between IMRT and 3D-CRT treatment planning

The patients were treated using IMRT technique (figure 4). Additionally, a 3D-conformal treatment planning with a four-field-technique (gantry angles 0°, 90°, 180°, 270°) was produced for each patient based on the identical CT datasets with the identical outlined CTV and OAR.

The comparison of minimum and maximum PTV doses between 3D-CRT and IMRT is shown in figure 5. IMRT minimum PTV dose ranged from 40 to 48.1 Gy, 3D-CRT minimum PTV dose from 47 to 49.3 Gy for the 2^nd ^order PTV. IMRT minimum PTV dose ranged from 62.4 to 67 Gy, 3D-CRT minimum PTV dose from 67.4 to 68.9 Gy for the 1^st ^order PTV. IMRT maximum PTV dose ranged from 72.4 to 74.2 Gy, 3D-CRT maximum PTV dose from 71.5 to 72.6 Gy for the 1^st ^order PTV.

**Figure 6:**
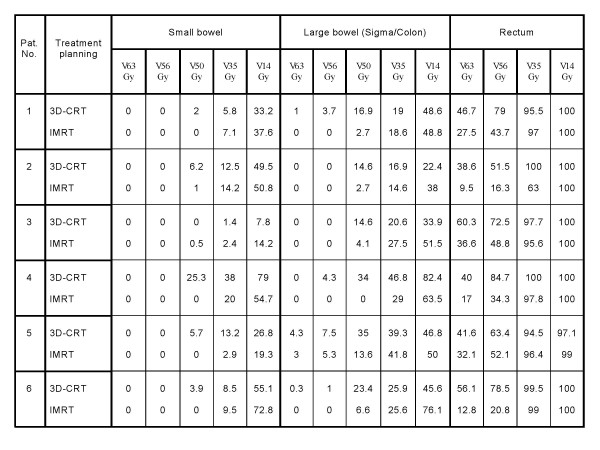
3D-CRT vs. IMRT. – DVH comparison of small bowel, large bowel and rectum. V63Gy, V56Gy, V50Gy, V35Gy, V14Gy denote the volume of a given organ (in %) irradiated with 63 Gy, 56 Gy, 50 Gy, 35 Gy, 14 Gy.

**Figure 7:**
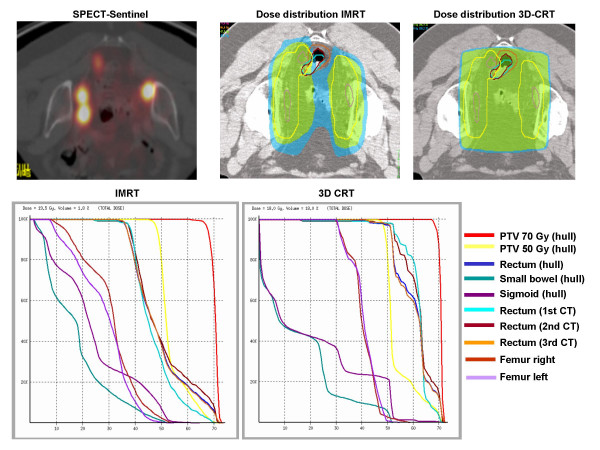
Planning example: Upper left : Sentinel lymph nodes left internal iliac (2), right internal iliac (1), perirectal on an axial SPECT image (prone position). Upper middle: IMRT planning: sentinel node localisations and second order PTV are outlined (prone position). The 95% isodose curve is shaped green. Upper right : 3D-CRT planning (four-field-box): sentinel node localisations and second order PTV are outlined (prone position). The 95% isodose curve is shaped green. Lower left: Dose-volume-histogram IMRT. Lower right: Dose-volume-histogram 3D-CRT

The DVH comparisons between IMRT and 3D-CRT in regard to small bowel, large bowel and rectum are shown in figure 6. Small bowel volumes irradiated with 14Gy and 35 Gy increased in four patients (in part very slightly) by using IMRT and decreased clearly in two patients with a volume reduction in more than 10%. With IMRT the critical dose to the small bowel of 50 Gy was seen in two patients with volumes of 0.5% and 1%, respectively, compared to 3D-CRT with five patients with volumes between 0 and 25.3%. Concerning the large bowel, IMRT similarly caused increased low dose irradiated volumes, but significant smaller irradiated volumes at the critical dose of 50 Gy. The most important difference was seen in the irradiated rectum volumes. All patients showed a clear benefit from IMRT with a clear decrease in volumes irradiated with 56 Gy and 63 Gy. As an example a dose distribution of a single patient are shown in figure 7.

### Acute toxicities

All patients completed the radiotherapy course after seven weeks with a dose of 70.0 Gy without treatment interruption. During radiotherapy interval, genitourinary toxicity RTOG Grade 1 occurred in all six patients, four patients increased to a genitourinary toxicity RTOG Grade 2. Gastrointestinal toxicity RTOG Grade 1 was seen in all six patients, two patients showed a gastrointestinal toxicity RTOG Grade 2. No gastrointestinal or genitourinary acute toxicity Grade 3 or 4 was noted. There were no toxicities seen concerning the skin. All patients reported erectile dysfunction, since neoadjuvant hormonal ablation had been started. After three months, two patients reported slight genitourinary symptoms (RTOG Grade 1), one of them had a slightly increased defecation frequency corresponding to a gastrointestinal toxicity RTOG Grade 1. The other four patients did not show any toxicity after three months.

## Discussion

Although being still controversial, several data suggest that an adjuvant coverage of the pelvic lymph nodes increases the likelihood of tumour control in patients with high risk prostate cancer. Whereas several older trials suggested that larger irradiation portals may not increase the treatment efficacy [32-41], recent randomised and nonrandomised studies could demonstrate a benefit of elective pelvic lymph node irradiation [21-23,42-47].

Of special value is the randomised four-armed RTOG 94-13 trial, since it was designed to provide a final answer regarding the role of adjuvant lymph node coverage. It showed that high risk prostate cancer patients benefit from radiotherapy of the pelvic lymph nodes combined with neoadjuvant and concurrent hormonal ablation. A significant benefit was documented in terms of advanced 4-year progression free survival rates [23], but not for overall survival. Interestingly, there was no significant difference in toxicities between patients treated on any of the four arms that may be caused by improved treatment techniques in comparison to former studies. The role of pelvic node coverage is also underlined by the fact that the EORTC trial [21] randomised high risk and lymph node positive patients to receive concurrent and long term adjuvant hormonal ablation treatment or radiotherapy alone, which included 50 Gy to the pelvis and a 20 Gy boost to the prostate. A substantial benefit in terms of local control, disease specific survival and overall survival was noted for the group receiving anti-hormonal treatment. Regarding 5-year clinical disease-free survival rates of 74% and 40% (no hormonal ablation) respectively, it has to be assumed that combined treatment with anti-hormonal therapy and radiation of the pelvic lymph nodes improves outcome of high risk and lymph node positive patients. Comparable results were shown by the RTOG 92-02 trial (including patients with cT2c-4, PSA < 150 ng/mL) combining radiotherapy of the pelvic nodes and the prostate with anti-hormonal short vs. long term treatment. An overall survival advantage was seen in a subset analysis of patients with Gleason Scores 8 to 10 (81% vs. 71%) [22].

Regarding efficacy, available data make it highly likely that the subgroup of patients with high risk of pelvic lymph node involvement will benefit from this treatment. Thus, it can be assumed that any further improvement in staging and irradiation techniques will help to optimise outcome. The sentinel concept was mainly developed for an optimised surgical lymph node identification and dissection in patients with malignant melanoma [48]. This concept can also be perfectly applied to breast cancer [49], head & neck cancer [50] and probably also to prostate cancer [24,25]. Key work was done by Wawroschek and Harzmann providing evidence that ^99m^Tc-Nanocoll based sentinel node identification is highly sensitive and specific. Similar results were obtained by us in a smaller cohort [51]. Therefore, sentinel image data may be used for radiation treatment planning.

The discussion on a potentially increased toxicity of larger irradiation portals was mainly supported by results from older studies, which were not based on modern CT planning. Thus, the favourable results from the RTOG 94-13 trial may in part be explained by optimised treatment techniques. Meanwhile, few clinical studies suggest superiority of IMRT when used for radiotherapy of the prostate alone [7]. However, even less data are available concerning an inclusion of the pelvic lymph nodes with IMRT. Nutting et al. [52] performed a theoretical trial in 10 patients and compared different IMRT treatment techniques among each other and to conventional 3D-CRT. The data provided in this study show eloquently that IMRT based irradiation protocols allow clear reduction of limiting normal tissue exposure. In a second paper from the same group, quality assurance aspects of clinical introduction comparing a step and shoot versus dynamic arch application were described. The authors concluded that the technique is feasible and neither of both application modi was superior [53]. Similarly, Mundt analysed the applicability of an IMRT based protocol for the treatment of pelvic lymph nodes in patients with gynaecological malignancies [54]. The authors conclude that IMRT based irradiation is associated with a diminished normal tissue toxicity profile and an optimised target coverage. However, in contrast to our work, both authors used anatomically based target volume definitions rather than an individualised sentinel lymph nodes based planning approach.

**Figure 8:**
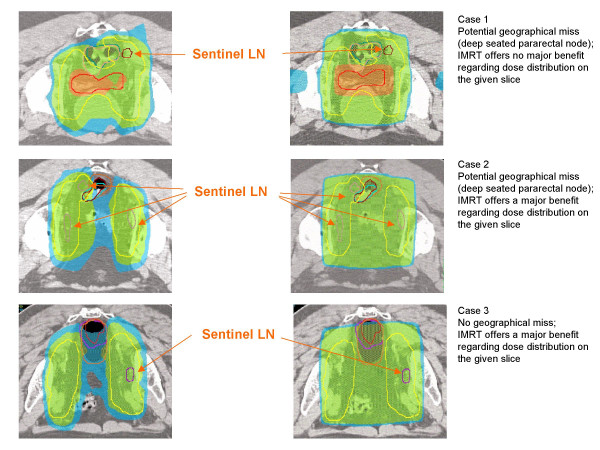
Axial CT slices of three example cases.

Our approach to combine sentinel data with IMRT addresses crucial aspects of target coverage and also toxicity. To reduce the likelihood of a 'geographical miss' we purposed the complete individualised coverage of lymphatic drainage using functional imaging. Possibly, individualised target volumes may cause increased toxicities. Not to mention, we observed a notable number of sentinel nodes nearby the dorsal rectal wall and their inclusion into the target volumes causes increased toxicities when a conventional four-field-technique is used (figure 8). Whereas the focus of 3D treatment planning is on target coverage, IMRT target coverage is compromised by the need of normal tissue sparing. Our own data hint at an optimal target volume coverage and showed a clear benefit in OAR sparing by using IMRT with low overall acute toxicities. Thus, IMRT is necessary to decrease toxicities resulting from individualised target volume definition.

## Conclusion

In order to optimise the balance between increased probability of side effects and improved target volume coverage, we tested a sentinel node based IMRT approach. Based on a limited number of patients the following conclusions can be drawn: Sentinel functional imaging optimised target volume definition for IMRT is clearly feasible with no obvious increase in acute toxicity and suitable for larger numbers of patients. The probability of a geographical miss in target volume definition might be reduced by an individualised target volume definition. However, larger series are necessary to verify the expected benefit in regard to efficacy and toxicities.

## Abbreviations

3D-CRT Three-dimensional conformal radiotherapy

CT Computed tomography

CTV Clinical target volume

DVH Dose volume histogram

Gy Gray

ICRU International Commission on Radiation Units and Measurements

IMRT Intensity modulated radiotherapy

MBq mega Becquerel

MV mega Volt

ng/mL nanogram/millilitre

OAR Organ at risk

PSA Prostate specific antigen

PTV Planning target volume

RTOG Radiation Therapy Oncology Group

SN Sentinel node

SPECT Single Photon Emission Computed Tomography)

TNM TNM stage classification (tumour/nodal/metastases stage)

Tc Technetium

vs. versus

## Competing interests

The author(s) declare that they have no competing interests.

## Authors' contributions

CB & UG planned, coordinated and conducted the study. KE, WB, FP took part in designing the study. UG, CB, MA, MBi analyzed the data. UG & CB prepared the manuscript. Medical care was covered by FP, SC, KE, SG, IH, AA, AS, RB, WB, MBa. All authors read and approved the final manuscript.

## Pre-publication history

The pre-publication history for this paper can be accessed here:
